# A role for RNA knots in Alzheimer’s disease

**DOI:** 10.7554/eLife.106119

**Published:** 2025-02-28

**Authors:** Silvia Galli, Marco Di Antonio

**Affiliations:** 1 Imperial College London London United Kingdom

**Keywords:** G-quadruplex, alzheimer's disease, aging, RNA, tau, APOE, Human

## Abstract

The buildup of knot-like RNA structures in brain cells may be the key to understanding how uncontrolled protein aggregation drives Alzheimer’s disease.

**Related research article** Kallweit L, Hamlett ED, Saternos H, Gilmore A, Granholm AC, Horowitz S. 2025. Chronic RNA G-quadruplex accumulation in aging and Alzheimer’s disease. *eLife* **14**:RP105446. doi: 10.7554/eLife.105446.

Memories are fundamental to any human being. They provide a sense of self-identity and shape our perception of the world and our loved ones. This is why neurogenerative diseases involving memory loss, like dementia, are highly debilitating, even when an individual’s physical fitness is not affected.

Alzheimer’s disease is the most common type of dementia, accounting for 60–70% of all cases, and it is projected that around 139 million will be suffering from the disease by 2050 (WHO, 2023). This means that a significant proportion of the world’s population will lose their precious memories in the foreseeable future, resulting in unsustainable pressure on the healthcare system. Given the lack of viable therapies currently available to treat Alzheimer’s, there is an urgent need to identify new molecular targets that can be leveraged to develop innovative therapeutic agents.

Although the exact mechanisms that lead to Alzheimer’s disease are not fully understood, it is well known that an uncontrolled build-up of insoluble protein aggregates in the brain is a hallmark of the disease. Amyloid-beta proteins have been shown to accumulate as clumps outside brain cells, whilst p-tau proteins clump to form neurofibrillary tangles that have been detected in the cytoplasm of neurons and other brain cells (Braak and Braak, 1995; Furman et al., 2017).

In this context, RNA molecules that stabilize protein aggregates are becoming increasingly relevant as potential therapeutic targets (Raguseo et al., 2023; Wolozin and Apicco, 2015). Recently, RNA structures called G-quadruplexes – known for their roles in gene expression and protein production – have emerged as key drivers of aggregate formation in brain cells affected by amyotrophic lateral sclerosis, raising the possibility that they may also be implicated in other neurodegenerative diseases, such as Alzheimer’s disease (Raguseo et al., 2023). Now, in eLife, Scott Horowitz and colleagues from the University of Denver, the Medical University of South Carolina, and the University of Colorado – including Lena Kallweit as first author – report that brain samples taken from Alzheimer’s disease patients show a significant accumulation of RNA G-quadruplex structures (Kallweit et al., 2025).

Kallweit et al. studied brain tissues from deceased people with Alzheimer’s disease aged between 30 and 92 years. Using a combination of immunostaining and fluorescence microscopy, the team showed that older patients had a higher number of RNA G-quadruplexes than younger patients. This connection became even stronger as the disease progressed. The prevalence of RNA G-quadruplexes further increased with the size of the area where p-tau aggregates accumulated.

Besides aging, genetic factors can also contribute to the development of Alzheimer’s disease. It is known that a specific variant of the gene APOE (known as APOE4) is linked to the accumulation of amyloid-beta aggregates (Cicognola et al., 2025). Taking this into account, Kallweit et al. showed that brain samples from patients with the APOE4 variant had a higher number of RNA G-quadruplexes than those without the variant. This suggests that genetic variations known to promote the accumulation of protein aggregates linked to Alzheimer’s disease could also potentially drive the accumulation of RNA G-quadruplex structures.

Taken together, the findings suggest that RNA G-quadruplexes may have an active role in neurodegeneration and support a model in which RNA G-quadruplexes could become tangled with p-tau proteins, leading to insoluble aggregates inside brain cells (Figure 1). This idea is further supported by previous research showing that RNA G-quadruplexes increase the aggregation of p-tau in experiments with cultured cells (Zwierzchowski-Zarate et al., 2022). This suggests that accumulating RNA G-quadruplexes in brain cells, especially when combined with genetic variations such as APOE4 and aging, may promote the formation of p-tau aggregates that drive Alzheimer’s disease.

**Figure 1.**
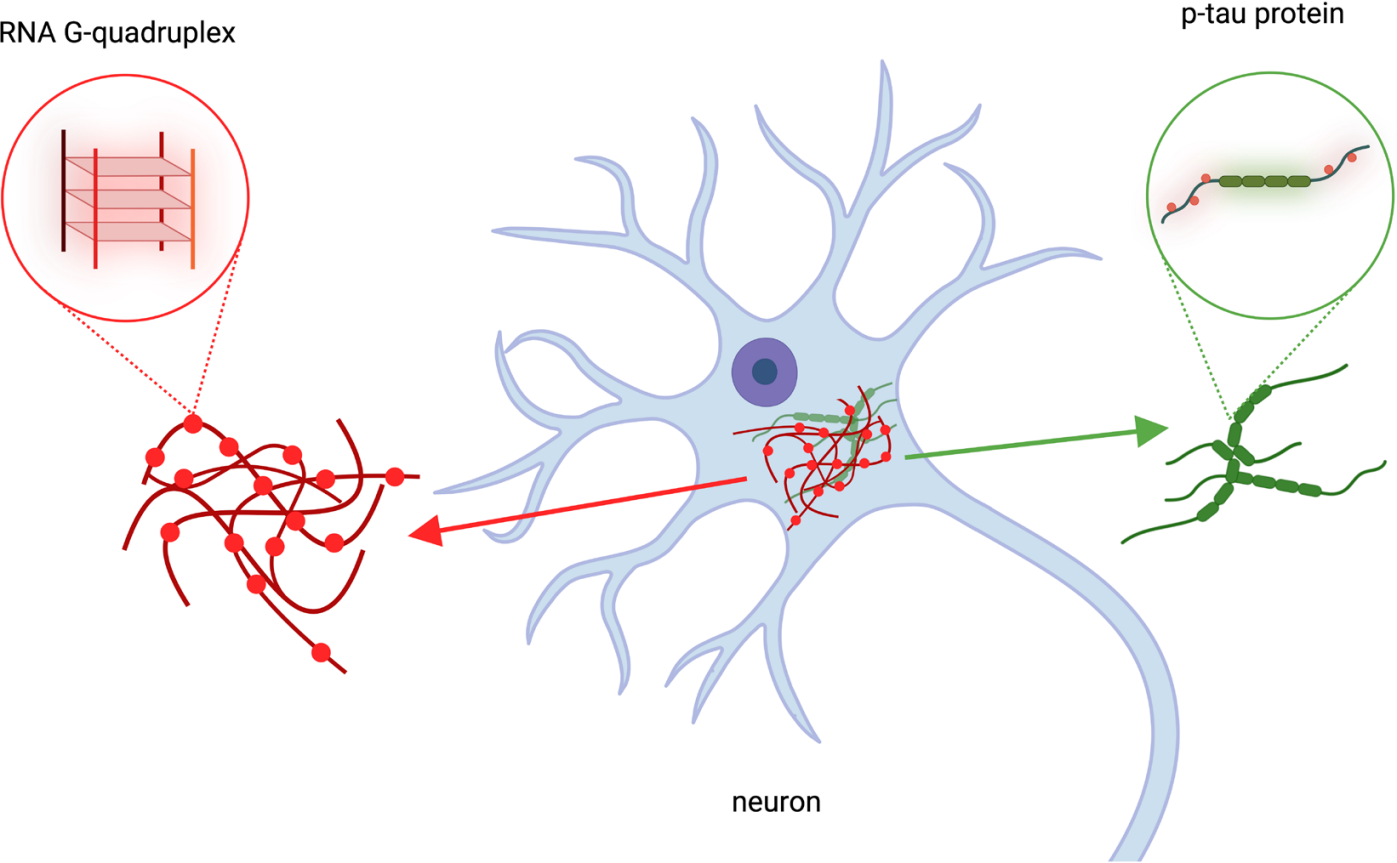
The role of RNA G-quadruplexes in Alzheimer’s Disease. Kallweit et al. analyzed brain cells taken from deceased patients with Alzheimer’s disease. This revealed that RNA structures called RNA G-quadruplexes (red structures, left), which help regulate gene expression, accumulate in the same location as p-tau protein clusters (green, right). Clumps of protein p-tau and RNA G-quadruplexes overlapped in brain cells (blue structure in the center). This figure was created using BioRender.com.

The results of Kallweit et al. further highlight the potential of targeting RNA – including G-quadruplexes – to diagnose or treat neurodegenerative diseases (Abad-Yang et al., 2025). Given the high correlation with the severity of Alzheimer’s disease, RNA G-quadruplexes could be used as a biomarker for early Alzheimer’s disease development to help accelerate diagnoses. RNA targeting and degradation have been successfully employed in other conditions, which may one day be a reality for Alzheimer’s disease (Tong et al., 2024). In the future, small molecules that can target and dissolve RNA complexes may be the key to treating incurable neurodegenerative conditions like Alzheimer’s.
